# Albumin-bound paclitaxel augment temozolomide treatment sensitivity of glioblastoma cells by disrupting DNA damage repair and promoting ferroptosis

**DOI:** 10.1186/s13046-023-02843-6

**Published:** 2023-10-28

**Authors:** Shanqiang Qu, Songtao Qi, Huayang Zhang, Zhiyong Li, Kaicheng Wang, Taichen Zhu, Rongxu Ye, Wanghao Zhang, Guanglong Huang, Guo-zhong Yi

**Affiliations:** 1 Department of Neurosurgery, Nanfang Hospital, Southern Medical University, Guangzhou, Guangdong People’s Republic of China; 2 The Laboratory for Precision Neurosurgery, Nanfang Hospital, Southern Medical University, Guangzhou, Guangdong People’s Republic of China; 3 Nanfang Glioma Center, Guangzhou, Guangdong People’s Republic of China; 4 Institute of Brain Disease, Nanfang Hospital, Southern Medical University, Guangzhou, Guangdong People’s Republic of China; 5 The First Clinical Medical College, Southern Medical University, Guangzhou, Guangdong People’s Republic of China

**Keywords:** Glioblastoma, Temozolomide sensitivity, Albumin-bound paclitaxel, DNA damage repair, Ferroptosis

## Abstract

**Background:**

Temozolomide (TMZ) treatment efficacy in glioblastoma (GBM) patients has been limited by resistance in the clinic. Currently, there are no clinically proven therapeutic options available to restore TMZ treatment sensitivity. Here, we investigated the potential of albumin-bound paclitaxel (ABX), a novel microtubule targeting agent, in sensitizing GBM cells to TMZ and elucidated its underlying molecular mechanism.

**Methods:**

A series of in vivo and in vitro experiments based on two GBM cell lines and two primary GBM cells were designed to evaluate the efficacy of ABX in sensitizing GBM cells to TMZ. Further proteomic analysis and validation experiments were performed to explore the underlying molecular mechanism. Finally, the efficacy and mechanism were validated in GBM patients derived organoids (PDOs) models.

**Results:**

ABX exhibited a synergistic inhibitory effect on GBM cells when combined with TMZ in vitro. Combination treatment of TMZ and ABX was highly effective in suppressing GBM progression and significantly prolonged the survival oforthotopic xenograft nude mice, with negligible side effects. Further proteomic analysis and experimental validation demonstrated that the combined treatment of ABX and TMZ can induce sustained DNA damage by disrupting XPC and ERCC1 expression and nuclear localization. Additionally, the combination treatment can enhance ferroptosis through regulating HOXM1 and GPX4 expression. Preclinical drug-sensitivity testing based on GBM PDOs models confirmed that combination therapy was significantly more effective than conventional TMZ monotherapy.

**Conclusion:**

Our findings suggest that ABX has the potential to enhance TMZ treatment sensitivity in GBM, which provides a promising therapeutic strategy for GBM patients.

**Supplementary Information:**

The online version contains supplementary material available at 10.1186/s13046-023-02843-6.

## Introduction

Glioblastoma multiforme (GBM) is the most common primary malignant brain tumor. According to statistics, it accounts for approximately 57.3% of all gliomas [1]. However, the prognosis for patients with GBM remains extremely poor, even when they receive aggressive comprehensive treatments. The median survival rate for GBM patients is less than 15 months, and the five-year survival rate is as low as 5% [1, 2]. Currently, the drugs available for clinical treatment of GBM are very limited, and temozolomide (TMZ) chemotherapy remains the only first-line drug used in clinics. Although TMZ treatment presents therapeutic potential for GBM patients in early stages, long-term use can lead to decreased sensitivity and even chemo-resistance [3, 4]. Therefore, improving the chemotherapeutic sensitivity of TMZ is currently a primary concern for clinicians, yet this issue has not been addressed in clinical practice.

TMZ is an alkylating agent that methylates guanine at the N7 position, adenine at the O3 position, and guanine at the O6 position. The cytotoxicity of this drug is mainly due to DNA damage induced by O6-methylguanine [5]. Our team previously reported that the nucleotide excision repair (NER) pathway, mediated by cytoskeleton-related protein DHC2, repairs TMZ-induced DNA damage in GBM cells. However, this repair mechanism ultimately leads to treatment failure in GBM [6]. Additionally, microtubules constitute a crucial component of the cytoskeleton in eukaryotic cells. Recent research has revealed that certain GBM cells interconnect via tumor microtubes (TM) to establish a network within the tumor, TMs exhibit the capacity to establish connections with fellow glioblastoma cells through gap junctions and adhesion junctions, further enhancing intercellular communication and may account for its resistance to various therapeutic interventions [7, 8]. The formation of synapses between neurons and tumor cells may contribute to the promotion of GBM proliferation through exploitation of the tumor network, although the precise underlying mechanism remains unclear [7]. Therefore, we hypothesized that the application of microtubule-targeting agents (MTAs) may enhance the sensitivity of GBM to TMZ.

Previous research has established that paclitaxel (PTX) exerts an anti-cancer effect by inhibiting tubulin depolymerization in various solid tumors [9, 10]. However, the clinical application of this treatment for GBM is severely limited by its inability to effectively penetrate the blood–brain barrier (BBB) [11]. Recently, a novel class of paclitaxel called albumin-bound paclitaxel nanoparticles (ABX) has been developed and reported to have the ability to penetrate the blood–brain barrier (BBB) [12, 13]. Consecutively, it has been demonstrated by Daniel Y Zhang et al. that ABX represents the optimal formulation for glioblastoma treatment due to its superior brain penetration and tolerability in comparison with traditional paclitaxel [14]. Additionally, microtubule-targeting agents (MTAs) have the potential to broadly suppress tumor cellular functions by disrupting microtubule connections between cells. In light of this, we postulate that ABX may enhance GBM sensitivity to TMZ.

In this study, we initially determined the in vitro concentration of TMZ and ABX combinations by calculating the IC50 values of individual drugs and utilizing the Chou-Talalay combination method. The synergistic anti-tumor effect of TMZ and ABX combinations was observed across multiple GBM cell lines, and subsequently validated in an intracranial orthotopic human GBM model. Subsequently, the impact of drug combinations on DNA damage and ferroptosis was assessed in GBM cells. In addition, this study further investigated the potential molecular mechanisms through LC–MS/MS analysis and validated them. Lastly, GBM patient-derived organoids (PDOs) were established to confirm the promising preclinical applications of the novel drug combination. The study offers a new perspective on the development of more effective therapies against GBM.

## Methods

### Cell line and GBM samples

The human U87-MG and LN229 cell lines used in this study were purchased from the American Type Culture Collection (ATCC). G353 and G393 are primary GBM cells isolated from fresh GBM tissues. Pathological information of primary cell patients was summarized in Table S1. All GBM samples were obtained from the Nanfang Glioma Center of Nanfang Hospital. GBM cells were cultured in Dulbecco's Modified Eagle Medium (Vivacell, C3113-0500) supplemented with 10% fetal bovine serum (BI Biotech, 04–001-1A) and maintained at 37℃ in a 5% CO_2_ incubator. This study received approval from the ethics committee of Nanfang Hospital, Southern Medical University.

### Cell viability assayand colony formation assay

Cell viability assays were performed in 96-well assays at 10^3^ cells per well culture (5 replicate wells per group) with indicated drug treatment for 72 h. Cell viability evaluation was executed by the Cell Counting Kit-8 (CCK-8) assay, which was performed using CCK-8 kit (Cat.no. C6005; New Cell & Molecular Biotech) according to the manufacturer’s instructions.

For the colony formation assay, GBM cells were cultured in six-well plates with 200 cells per well in the presence or absence of indicated drug treatment for 24 h. Later, the medium was replaced with fresh growth medium. After 2 weeks, visible colonies were fixed with 100% methanol and stained with 0.1% crystal violet in 20% methanol for 15 min. The area of colonies was calculated using ImageJ software (1.48v, National Institutes of Health, USA).

#### *Intracranial GBM-bearing mouse model and* in vivo analyses

The intracranial GBM-bearing mouse model was used in this study. All male nude mice (4–5 weeks old, BALB/c) were purchased from Sibeifu Biotechnology Co., Ltd. (Beijing, China). Briefly, the GBM cells were digested with 0.25% Trypsin–EDTA (Cat.no. C100C1; New Cell & Molecular Biotech), centrifuged and washed three times with 1xPBS. After successful anesthesia, the mice were fixed using brain stereotaxic instruments (Beijing Zhongshi Dichuang Technology Development Co., Ltd.), and conventional skin disinfection was performed. An incision was made in the skin on the skull and a burr hole was drilled. A ten microliter Hamilton syringe wasused for intracranial injection. Intracranial injections and skin suture closure were carried out as previously described [6]. All animal experiments were conducted with the approval from the Southern Medical University Institutional Committee for Animal Research and conformed with the national guidelines for the care and use of laboratory animals. Intracranial tumor growth was monitored in vivo in isoflurane-anaesthetized mice after inoculation using the Small Animal Imaging Facility (Bruker, FX Pro).

### Histology, immunohistochemistry and immunofluorescence

GBM samples, nude mouse tumor tissues and GBM organoids were fixed in 10% formalin for 1 h, washed, and suspended in 70% ethanol. Histology and immunohistochemistry were performed, details are provided in the supplementary methods. For immunofluorescence, 5 × 10^3^ cells were grown on 20 mm confocal petri dishes and received indicated drug treatments for 24–72 h. Images were captured on a Carl-Zeiss confocal microscope. Zen Black software (Zeiss) and ImageJ were used for image capture and analysis, respectively.

### Comet assay

The comet assay was performed with minor modifications as described [15]. The steps were performed according to kit instructions (WLA123; WanleiBio, Shenyang, China). All samples were stained and placed under the fluorescence microscope for observation. Data was analyses using the Comet Assay Software Project (CASP software).

### Ferroptosis assay

For detecting the ferroptosis status of GBM cells after receiving indicated drug treatments. The total quantities of glutathione were measured using a GSH Assay Kit (Dojindo, G263), Intracellular chelatable iron was determined using the fluorescent indicator FerroOrange (Dojindo, F374). Intracellular lipid peroxides were determined using a Liperfluo-detection kit (Dojindo, L248).

### GBM organoids construction and culture

Tumor tissue was collected from the operating room, suspended in ice cold culture media and brought to the lab on ice within 30 min from explantation. The protocol for constructing GBM organoids was referenced as previously described in the literature [16]. GBM organoids were plated on six-well, ultra-low adhesion plate (Corning; 3471). Plates were rotated at 120 rpm in a humidified incubator at 37°C, 5% CO2, and 21% oxygen. Short-Term GBM Organoid Mediumwas refreshed in organoid cultures every 48 h.

### Statistical analysis

Statistical analysis of SPSS 23.0 was used in this study. Difference between groups were determined by the independent student’s t test or analysis of variance (one- or two-way ANOVAs). For all tests, *P* values less than 0.05 were considered to be statistically significant.

## Results

### Combination therapy of TMZ and ABX displayed a significant synergistic effect on GBM cells

The viability of GBM cells was assessed by CCK-8 assays to determine the effects of TMZ and ABX. In this study, four GBM cell lines, namely U87-MG, LN229 and two primary GBM cell lines (G353 and G393), were selected for validation. The median inhibitory concentration (IC50) of TMZ and ABX was measured to determine the optimal drug combination. The concentration–response curves demonstrate that the IC50 values of TMZ in U87-MG, LN229, G353 and G393 cell lines were 673.69 µM, 1058.43 µM, 712.64 µM and 1122.52 µM respectively (Figure S1a). The IC50 values of ABX in U87-MG, LN229, G353 and G393 cell lines were 62.07 nM, 73.04 nM, 53.58 nM and 25.30 nM, respectively (Figure S1b). Overall, the sensitivity of GBM cells to the two drugs differed; ABX exhibited greater efficacy. Recognizing the side effects associated with the combination therapy of ABX and TMZ, we prudently chose a relatively low concentration of ABX (12 nM, approximately an IC_25_-IC_30_ concentration) and TMZ to conduct the following experiments in GBM cells. In U87 and G353 cells, we selected TMZ concentrations of 400µM corresponding to IC_25_ to IC_30_ levels, while for LN229 and G393 cells, we chose TMZ concentrations of approximately 800µM corresponding to IC_25_ to IC_30_ levels.

**Fig. 1:**
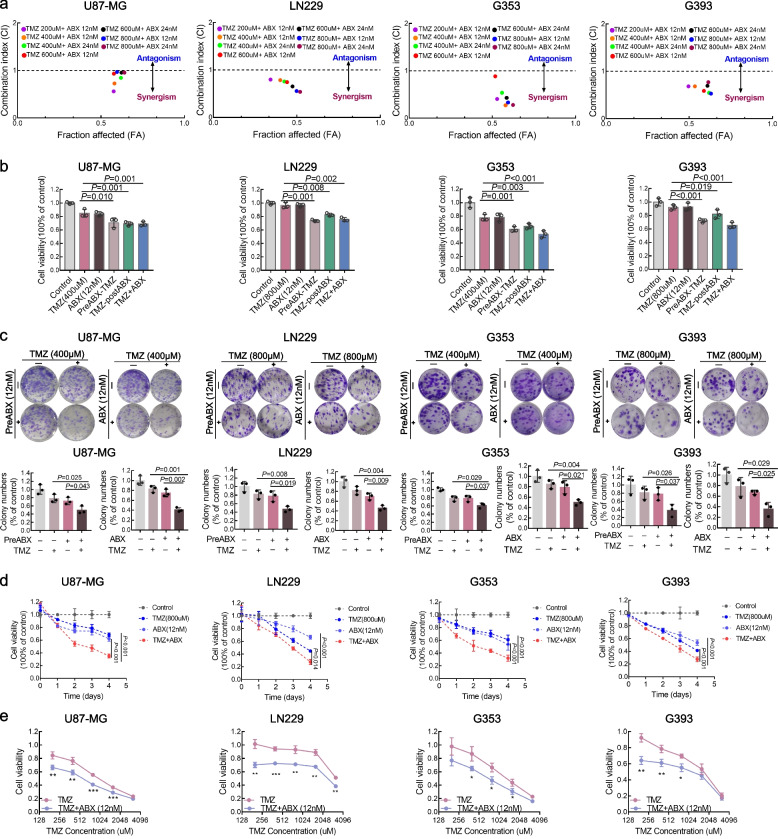
Combination therapy of TMZ and ABX displayed a significant synergistic effect on GBM cells. **a** The synergism of drug combination was calculated by using the Chou–Talalay Index. CI values less than, equal to, or greater than 1 indicate synergistic, additive, or antagonistic effects, respectively. **b** GBM cell viability was assessed using the CCK-8 assay after treatment with drugs at indicated concentrations for 48 h. Cultured cells were divided into six groups: the "Control" group, which was not exposed to TMZ or ABX for 48 h; the "TMZ" group, which received TMZ treatment for 48 h; the "ABX" group, which received ABX (12 nM) treatment for 48 h; the "preABX-TMZ" group, where cells received ABX (12 nM) treatment for 24 h followed by TMZ treatment for another 24 h. Conversely, the "TMZ-postABX" group was treated with TMZ for 24 h followed by ABX (12 nM) treatment for the subsequent 24 h. Finally, the “TMZ + ABX” group involved both treatments simultaneously. **c** The colony formation assay was performed on GBM cells treated with indicated drug concentrations for 48 h. **d** Cell viability of GBM cells was assessed with the indicated concentration of drug treatment on day 0, 1, 2, 3, and 4. **e** Cell viability of GBM cells was assessed after treatment with various concentrations of TMZ (200, 400, 800, 1600 and 3200 µM) for 72 h, with or without the addition of ABX (12 nM)

The sequential or concurrent treatments were two common clinical combination therapeutic approaches to maximizing drug-efficacy in clinic. To determine the synergistic effects of TMZ and ABX, rather than additive or antagonistic effects, we utilized CompuSyn software to calculate the combination index (CI) based on Chou-Talalay methodology [17]. The combination index (CI) values less than 1 indicate a synergistic effect, a CI equal to 1 indicates an additive effect, and a CI greater than 1 indicates antagonism between the two agents. Our findings indicated that the combination of TMZ and ABX resulted in a CI value less than 1 in GBM cells at specified concentrations (Fig. 1a and Figure S2), implying a synergistic effect between ABX and TMZ.The next step involves investigating the impact of the combined administration of two drugs and their combination mode on GBM cells. For this purpose, we have designed six distinct experimental groups, comprising of a control group, TMZ alone group, ABX alone group, preABX-TMZ group (sequential therapy group with ABX pre-treatment for 24 h before TMZ treatment), TMZ-postABX group (sequential therapy group with ABX post-treatment for 24 h after TMZ treatment) and the concurrent therapy group (TMZ + ABX).As depicted in Fig. 1b, the CCK-8 assay demonstrated that the preABX-TMZ group and the concurrent ABX treatment with TMZ group both significantly reduced GBM cell viability compared to TMZ monotherapy. To further validate the anti-tumor effect, we also conducted a colony formation assay. The results were consistent with those of the CCK-8 assays mentioned above, showing a significant decrease in colony numbers in both preABX-TMZ and TMZ + ABX groups compared to other groups (Fig. 1c). Furthermore, both preABX-TMZ and TMZ + ABX groups exhibited typical morphological features of cell death, including cytoplasmic contraction, cellular debris, and floating cells under microscopic observation at 48 h (Figure S3). It is noteworthy that the combination of TMZ and ABX exhibited a significant time-dependent inhibition on cell viability in U87-MG, LN229, G353 and G393 (Fig. 1d). Subsequently, we investigated the combined treatment effects of low-dose ABX (12 nM) and varying concentrations (200–3200 µM) of TMZ. As anticipated, the combination of TMZ and ABX exhibited a potent synergistic anti-tumor effect at different concentrations of TMZ (Fig. 1e and Figure S4). Collectively, these findings suggest that both sequential and concurrent administration of antibiotics can sensitize GBM cells to TMZ therapy. However, the concurrent treatment regimen of TMZ with ABX is superior to sequential treatment. The combinative treatment of ABX and TMZ exerts anti-tumor effects mainly by suppressing cell viability, inhibiting cell proliferation, and inducing cell death.

### Combination therapy of ABX and TMZ exhibited a significant inhibitory effect on the progression of GBM in vivo

**Fig. 2:**
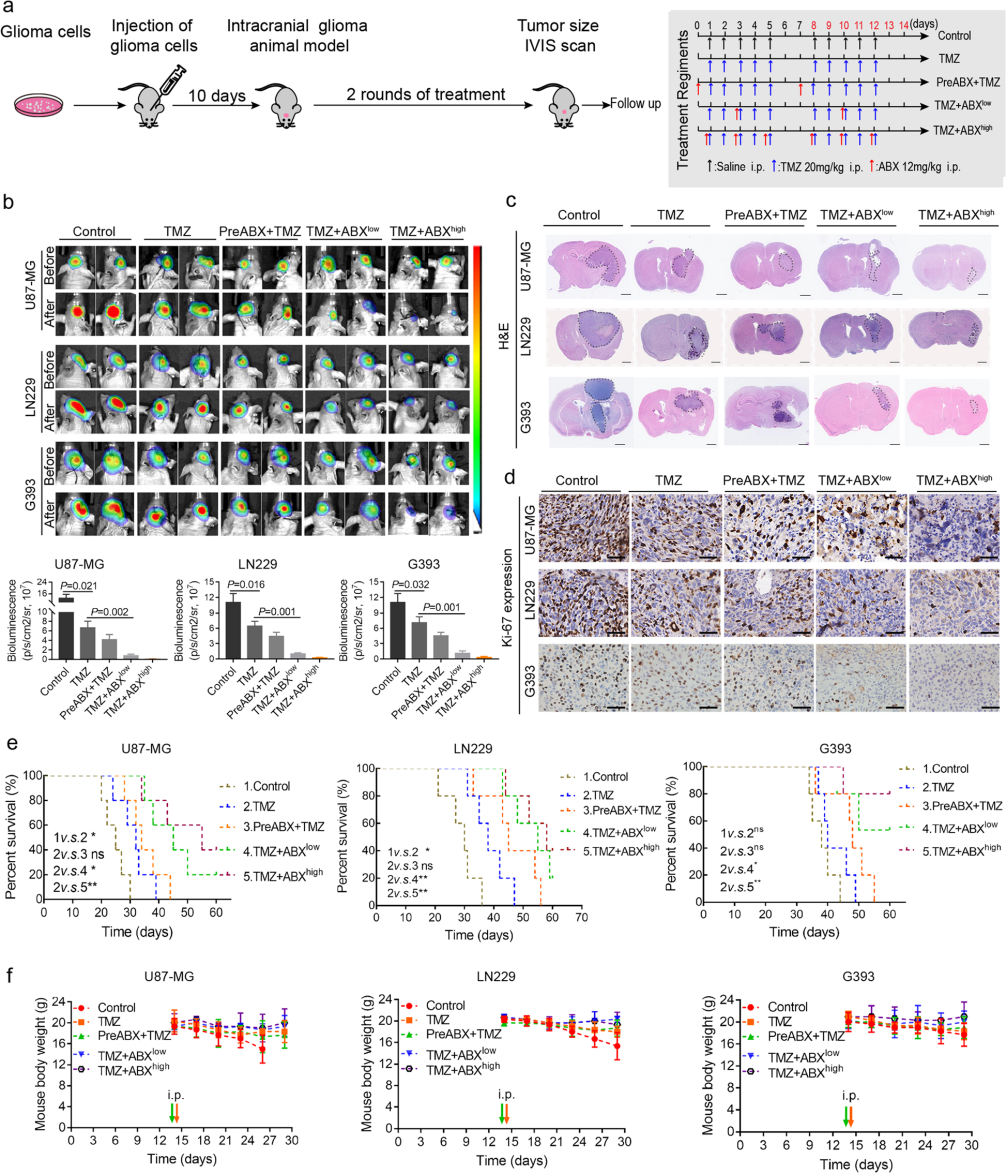
Combination therapy of ABX and TMZ exhibited a significant inhibitory effect on the progression of GBM in vivo. **a** The scheme involves intracranial orthotopic inoculation of glioma cells followed by systemic treatment regimens after tumor formation. Experimental nude mice were randomly divided into five groups, including Control (*n* = 5), TMZ alone (*n* = 5), preABX + TMZ (sequential therapy group with ABX pre-treatment for 24 h before TMZ treatment, *n* = 5), TMZ + ABX^low^ (concurrent therapy group with ABX treatment once per week, *n* = 5), and TMZ + ABX^high^ group (concurrent therapy group with ABX treatment three times per week, *n* = 5). Drug treatment was maintained for two cycles. **B** Top, the intracranial fluorescence images of GBM-bearing mouse models before and after drug treatment. Bottom, the intracranial fluorescence intensity statistics for each group after drug treatment. **c**-**d** Typical images of H&E staining (**c**) and Ki-67 immunohistochemical staining (**d**) are shown in orthotopic tumor tissue slices from GBM-bearing mice of each group. Scale bar, 50 µm. **e** A Kaplan–Meier survival curve was performed to analyze the survival prognosis of nude mice in each group with the indicated drug treatment. **f** Body weight changes in GBM-bearing mice were recorded for each group during the drug treatment period

To further validate the anti-tumor efficacy of TMZ and ABX combination therapy in vivo, an intracranial orthotopic GBM model was established in nude mice through implantation of luciferase-transfected GBM cells (U87-MG, LN229 and G393). The scheme for intracranial orthotopic inoculation of glioma cells and systemic treatment regimens following tumor formation are illustrated in Fig. 2a. Experimental nude mice were randomly assigned to five groups: Control, TMZ alone, preABX + TMZ (sequential therapy group with ABX pre-treatment for 24 h before TMZ treatment), and two concurrent therapy groups receiving either low or high frequency of ABX treatment in combination with TMZ. Intracranial fluorescence signals were initially observed and detected in each group following the successful establishment of an intracranial orthotopic GBM model, prior to treatment initiation. Following two weeks of drug therapy, intracranial fluorescence signals were once again observed and detected. As depicted in Fig. 2b, the combination therapy of TMZ and ABX resulted in a significant reduction of intracranial fluorescence intensity compared to both the control group and the TMZ treatment alone group. Compared to the sequential therapy group (preABX + TMZ), the concurrent therapy group (ABX + TMZ) also exhibited a significant reduction in intracranial fluorescence intensity following combinational treatment, particularly in the "TMZ + ABX^high^" subgroup. The representative H&E staining images of intracranial tumors in each group post-treatment are presented in Fig. 2c, and the findings were consistent with those depicted in Fig. 2b. Furthermore, Ki-67 IHC staining was performed in each group. The results demonstrated a significant decrease in Ki-67 expression in the combinational treatment groups compared to both the control and TMZ groups (Fig. 2d).

To further evaluate the impact of combined ABX and TMZ treatment on overall survival and adverse events in tumor-bearing nude mice, we monitored their survival time and body weight. Kaplan–Meier survival curves demonstrated that only the concurrent therapy regimen, and not the sequential therapy regimen, significantly prolonged the survival time of tumor-bearing nude mice. The "TMZ + ABX^high^" group exhibited the most favorable prognosis (Fig. 2e). The body weight of nude mice in each group was recorded and presented in Fig. 2f. The results showed no significant decrease after ABX administration compared with the TMZ alone group. Furthermore, we conducted histological analysis of the lungs, liver, kidneys, and spleen using H&E staining. The results showed no abnormal pathological changes in the combinational treatment group, indicating that there was no significant increase in toxicity after additional ABX treatment (Figure S5).

### Combination therapy of TMZ and ABX potentiated DNA damage and impeded DNA damage repair in GBM cells

**Fig. 3:**
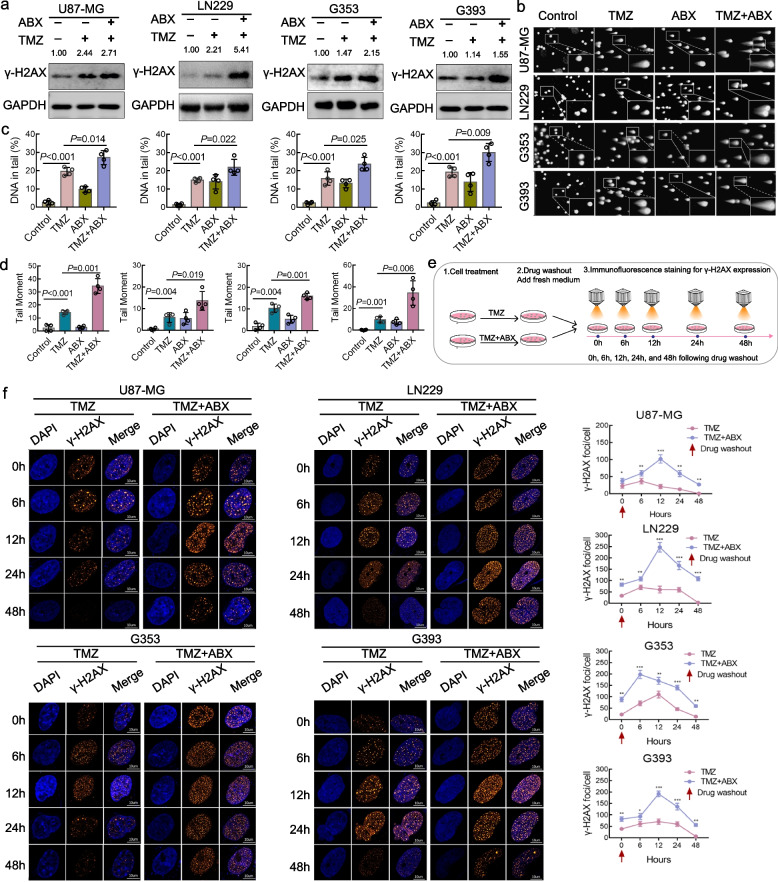
Combination therapy of TMZ and ABX potentiated DNA damage and impeded DNA damage repair in GBM cells. **a** Protein levels of γ-H2AX were analyzed by western blotting in GBM cells treated with TMZ (400 µM for U87-MG and G353, 800 µM for LN229 and G393) with or without ABX (12 nM) for 48 h. **b**-**d** Typical comet assay images of GBM cells treated with the indicated drugs for 48 h in each group (**b**), as well as quantification and statistical analysis of DNA in the tail (**c**) and tail moment (**d**). **e** Schematic illustration of dynamic detection of γ-H2AX foci at various time points after drug treatment is indicated to monitor the course of DNA damage repair. **f** Left, dynamic changes of γ-H2AX foci were observed in GBM cells treated with TMZ with or without ABX at various time points following drug elution. Right, statistical analysis was conducted on the number of γ-H2AX foci in GBM cells at various time points following drug elution. Scale bar, 10 µm

Previous research has established that the byproducts of TMZ decomposition can disrupt DNA replication and induce DNA damage in GBM cells. However, the robust DNA damage repair system and intricate repair mechanisms in GBM cells are significant factors contributing to the resistance of TMZ. We investigated the DNA damage response status in GBM cells treated with a combination of TMZ and ABX, including both sequential and concurrent therapy. The western blotting results demonstrated that both sequential and concurrent application of TMZ and ABX treatment significantly increased the expression of γ-H2AX protein in GBM cells compared to TMZ mono-treatment and control group (Fig. 3a and Figure S6). We also conducted comet assay to detect DNA damage in individual cells. The results revealed that the combination of TMZ and ABX treatment significantly induced DNA damage in GBM cells, as evidenced by an increase in the percentage of DNA in tail and tail moment (Fig. 3b, c, and d). In addition, we conducted immunofluorescence and confocal microscopy scanning to analyze the levels of γ-H2AX expression in GBM cells. The results were consistent with those obtained from western blotting (Figure S7), indicating that combination treatment of TMZ and ABX could enhance DNA damage. Further, we also evaluated the capacity of DNA damage repair in GBM cells following the indicated treatment. To monitor the progress of DNA damage repair, GBM cells were treated with TMZ or TMZ + ABX for 48 h. After drug elution, the cells were maintained in normal culture media and γ-H2AX expression in the nucleus was detected by immunofluorescence at different time points (0, 6, 12, 24 and 48 h) following drug removal (Fig. 3e). The results indicate that the majority of DNA damage was repaired within 48 h after drug washout in the TMZ mono-treatment group. However, in the TMZ plus ABX treatment group, γ-H2AX levels were sustained above basal level for a longer period compared to the TMZ mono-treatment group, indicating persistent DNA damage (Fig. 3f). The aforementioned findings were further confirmed through γ-H2AX immunohistochemical staining in vivo. The findings validated that the combination therapy of TMZ and ABX significantly augmented γ-H2AX expression in comparison to other groups (Figure S8).

### Combination therapy of TMZ and ABX enhanced DNA damage by disturbing expression and nuclear translocation of DNA repair proteins ERCC1 and XPC

**Fig. 4:**
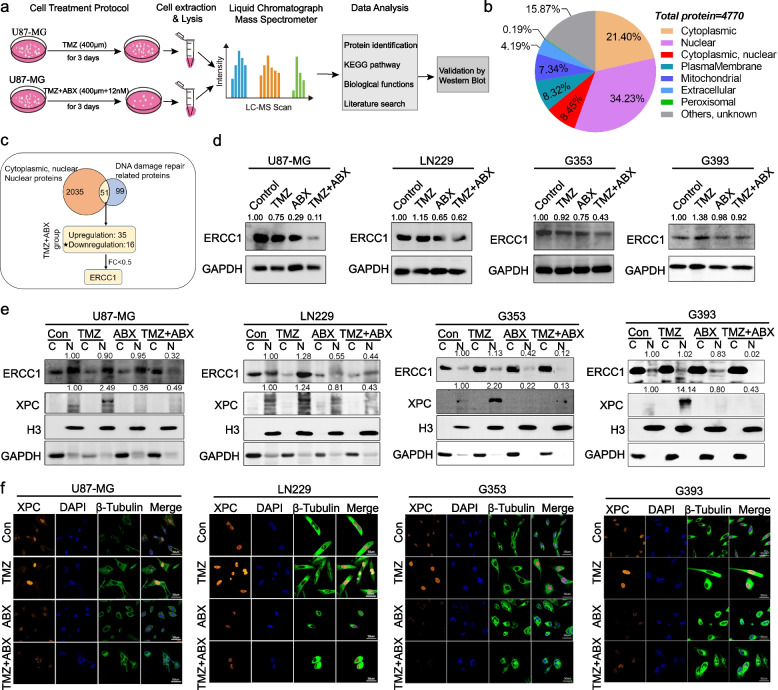
Combination therapy of TMZ and ABX enhanced DNA damage by disturbing expression and nuclear translocation of DNA repair proteins ERCC1 and XPC. **a** The schematic diagram illustrates the proteomics design and analysis process, which aims to further explore the potential molecular mechanisms of drug combination treatment. **b** The analysis of total number and subcellular components of proteins identified in proteomics. **c** The schematic diagram of downstream target proteins selection. To explore the potential molecular mechanism of enhanced DNA damage in drug combination treatment group, we mainly focused on nuclear localization proteins and DNA damage repair related proteins. A total of 51 potential target proteins were identified. Furthermore, in order to investigate the down-regulated DNA damage-related proteins underlying combination therapy, data screening was conducted based on a fold change standard (TMZ + ABX vs. TMZ) of less than 0.5. Finally, we screened out ERCC1 protein. **d** The total expression of ERCC1 protein in GBM cells from each group with the indicated drug treatment was assessed by western blotting. **e** Western blotting analysis was performed to examine the expression of ERCC1 and XPC proteins in cytoplasmic (C) and nuclear (N) fractions of GBM cells after drug treatments. GAPDH and Histone H3 were used as internal loading controls. **f** Immunofluorescence staining was performed to detect the localization of XPC protein in GBM cells treated with TMZ (400 µM for U87-MG and G353 cells; 800 µM for LN229 and G393 cells) and ABX (12 nM)

To further investigate the potential molecular mechanism underlying the increased DNA damage observed in the drug combination treatment group, we utilized LC–MS/MS to compare alterations in cellular protein expression between U87-MG cells treated with TMZ alone and those treated with both TMZ and ABX (Fig. 4a). The cellular morphological changes and γ-H2AX protein expression verification between the two groups are presented in Figure S9. The mass spectrometry analysis revealed the identification of a total of 4770 proteins in U87-MG cells, with 21.40% localized in the cytoplasm, 34.23% in the nucleus, and 8.45% present in both compartments (Fig. 4b). Since DNA damage repair pathways occur in the cellular nucleus, our focus is primarily on proteins located within the nucleus (*n* = 2035). After intersecting 2035 nuclear-localized proteins with 150 reported DNA damage repair-related proteins [18], a total of 51 potential target proteins were identified. Furthermore, in order to investigate the down-regulated DNA damage-related proteins underlying combination therapy, data screening was conducted based on a fold change standard (TMZ + ABX *vs.* TMZ) of less than 0.5. Finally, we identified the ERCC1 protein as a crucial component of the nucleotide excision repair pathway (NER) (Fig. 4c). In our previous research, we have demonstrated that the cytoskeletal-related protein DHC2 facilitates nuclear transport of XPC, another crucial NER-associated protein, to repair DNA damage induced by TMZ and ultimately mediate acquired resistance in GBM [6]. To achieve this goal, we hypothesized that the combination of ABX and TMZ could induce persistent DNA damage by modulating the expression and nuclear translocation of critical proteins (such as ERCC1 and XPC) in the NER pathway.

Subsequently, we performed western blotting and immunofluorescence assays to further substantiate our hypothesis. The total, nuclear, and cytoplasmic proteins of GBM cells were extracted following the indicated treatments. The western blot results demonstrated a reduction in total ERCC1 protein expression in the TMZ plus ABX treatment group compared to both mono-treatment and control groups (Fig. 4d). The subcellular distribution analysis revealed a significant reduction in nuclear expression of ERCC1 and XPC proteins following treatment with TMZ plus ABX, as compared to mono-treatment and control groups (Fig. 4e). The results of immunofluorescence staining demonstrated that XPC protein accumulated in the nucleus following treatment with TMZ, but was absent in both ABX and TMZ plus ABX groups (Fig. 4f).

### Combination therapy of TMZ and ABX induced ferroptosis of GBM cells by regulating HOXM1 (HO-1) and GPX4 expression

**Fig. 5:**
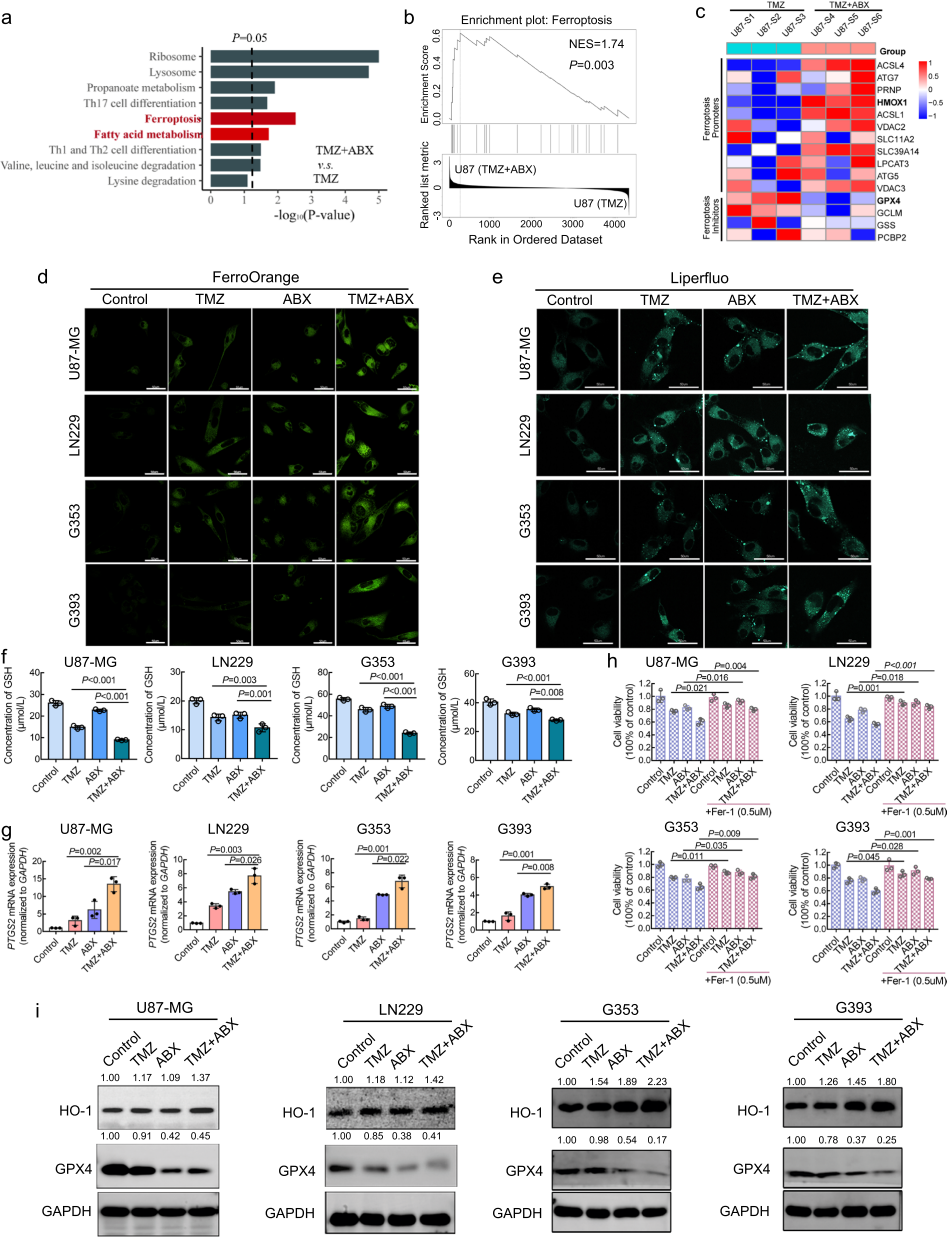
Combination therapy of TMZ and ABX induced ferroptosis of GBM cells by regulating HOXM1 (HO-1) and GPX4 expression. **a** KEGG pathway enrichment analysis was performed based on the different expressed proteins identified in proteomics research. **b** Gene set enrichment pathway analysis (GSEA) showed enrichment of ferroptosis signaling pathway in TMZ plus ABX treatment group. **c** Protein expression heatmap of the ferroptosis-promoted and inhibited proteins in U87-MG cells treated with TMZ (400 µM) with or without ABX (12 nM) according to LC–MS/MS data. **d**-**e** Representative confocal fluorescence microscopy images of iron staining with ferroOrange (**d**) lipid-ROS staining with liperfluo (**e**) in GBM cells with indicated treatments for 72 h. Scale bar, 50 µm. **f** Relative glutathione levels in GBM cells with indicated treatments for 72 h. **g** Relative mRNA levels of PTGS2 in GBM cells with indicated treatments for 72 h. **h** Cell viability of GBM cells treated with indicated concentrations of TMZ or ABX, and with or without addition of ferrostain-1 (2 µM, ferroptosis inhibitor). **i** Protein expression level of HO-1 and GPX4 were analyzed by western blotting in GBM cells with indicated treatment for 72 h

Further, we also performed bioinformatic analysis on the differentially expressed proteins between the TMZ monotherapy group and the combined TMZ + ABX therapy group. Notably, the KEGG pathway analysis results demonstrated a significant enrichment of the terms "Ferroptosis" and "Fatty acid metabolism" (Fig. 5a). The upregulation of the ferroptosis pathway was also confirmed in the TMZ plus ABX treatment group through GSEA analysis (Fig. 5b). The heatmap of protein expression based on proteomic data revealed that the proteins promoting ferroptosis were upregulated, while those inhibiting ferroptosis were downregulated in the combination treatment group compared to the TMZ mono-treatment group (Fig. 5c).

Ferroptosis is a newly identified form of cell death that results from iron-dependent lipid peroxidation. We employed liperfluo, Ferrous iron (Fe^2+^), and Glutathione (GSH) detection kits, the most commonly utilized indicators for ferroptosis detection, to assess the ferroptosis status of each group. For live cell imaging, we observed a significant accumulation of Fe^2+^ (Fig. 5d) and lipid-reactive hydrogen peroxide (Fig. 5e) in the cytoplasm of GBM cells following combination treatment with TMZ and ABX, as compared to mono-treatment with TMZ. The combination treatment of TMZ and ABX resulted in a significant reduction in intracellular GSH levels compared to mono-treatment (Fig. 5f). Additionally, we have also identified the mRNA expression level of the PTGS2 gene, which has recently been established as a standard marker for ferroptosis in vitro [19]. The combination treatment resulted in a significant upregulation of PTGS2 mRNA levels in GBM cells compared to mono-treatment (Fig. 5g), as revealed by qRT-PCR analysis. Compared to TMZ mono-treatment, the viability of GBM cells was significantly reduced after 48 h of treatment with TMZ plus ABX. However, addition of a ferroptosis inhibitor, Fer-1, partially rescued GBM cell viability in the combination treatment group (Fig. 5h), suggesting that the combination therapy of TMZ plus ABX could induce ferroptosis in GBM cells and that ferroptosis may represent only a portion of the anti-tumor effects mediated by this combination. Next, we investigated the potential crosstalk between ferroptosis and DNA damage induced by combination therapy. Our findings from immunofluorescence and western blotting analyses revealed that Fer-1 treatment did not affect γ-H2AX expression or the number of γ-H2AX foci in GBM cells treated with TMZ plus ABX, indicating an absence of crosstalk between ferroptosis and DNA damage induced by combination therapy (Figure S10).

However, the molecular mechanism underlying ferroptosis induced by combination treatment remains unclear. From the proteomics data, we have observed a significant fold change in the levels of GPX4 and HO-1 proteins between TMZ and TMZ + ABX groups. Increasing evidence has confirmed the significant roles of GPX4 and HO-1 proteins in inducing ferroptosis. Subsequently, western blotting was performed to examine the expression levels of GPX4 and HO-1 proteins, revealing a significant downregulation of GPX4 expression and a significant upregulation of HO-1 expression in GBM cells treated with combination therapy compared to other groups (Fig. 5i). The aforementioned findings were further confirmed through HO-1 and GPX4 immunohistochemical staining in vivo. The findings validated that the combination therapy of TMZ and ABX significantly augmented HO-1 and reduced GPX4 expression in comparison to other groups (Figure S8).

### Preclinical assessment of the combined therapeutic efficacy of TMZ and ABX in patient-derived GBM organoid models

**Fig. 6:**
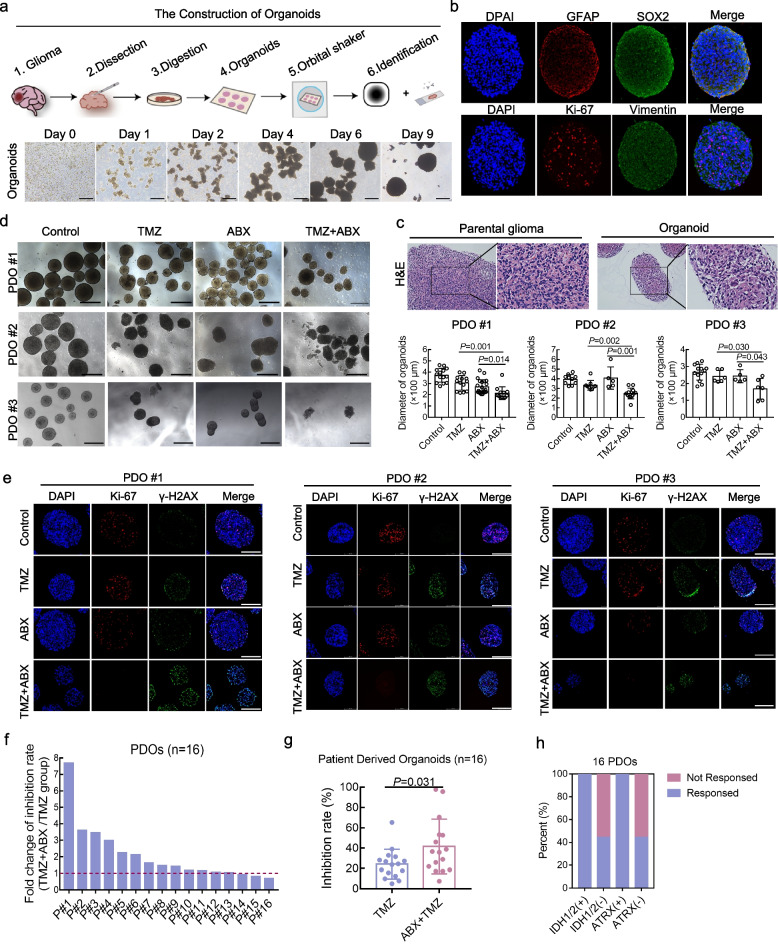
Preclinical assessment of the combined therapeutic efficacy of TMZ and ABX in patient-derived GBM organoid models. **a** Top, schematic illustration of PDOs construction. Bottom, representative images of GBM PDOs culture process. Scale bar, 400 µm. **b** The GFAP, SOX2, Ki-67 and Vimentin markers were detected in tissue slices of GBM PDOs by immunofluorescence staining. **c** Representative H&E staining images of GBM PDOs and parental GBM tumor tissue slices are presented. **d** Representative images of GBM PDOs after indicated drug treatments for 5 days, the morphology and size of the organoids were observed and analyzed. Scale bar, 400 µm. **e** Immunofluorescence staining of Ki-67 and γ-H2AX was conducted to evaluate the PDOs proliferation index and DNA damage index in each treatment group. **f**-**g** The ratio of inhibition rate was calculated for 16 GBM patient-derived organoids (PDOs) treated with a combination of TMZ and ABX, compared to those treated with TMZ alone. **h** GBM PDOs were classified based on IDH1/2 and ATRX mutations, and the respective efficacy of TMZ and ABX combination therapy was quantified for each group

Patient-derived organoids (PDOs) have demonstrated significant potential in advancing drug screening and predicting drug sensitivity for cancer treatment. To evaluate the clinical therapeutic potential of TMZ and ABX combination treatment, we have successfully generated several GBM PDOs. The schematic diagram depicting the construction of GBM organoids and the dynamic process of their culture is presented in Fig. 6a. The majority of PDOs can be successfully generated within a two-week timeframe, achieving diameters ranging from 400 to 600 µm. Subsequently, H&E staining was conducted on both PDOs and their corresponding parental tumors, revealing strikingly similar histological features of GBM between the two tissues (Fig. 6c and Figure S11a). We also conducted immunofluorescence staining for glial markers GFAP, glioma stem cell markers SOX2, tumor proliferation marker Ki-67 and invasion markers Vimentin. The expression of related markers was observed in all GBM PDOs (Fig. 6b and Figure S11b).

Furthermore, we evaluated the antitumor efficacy and DNA damage response in three distinct GBM PDOs models following indicated treatments. The PDOs were observed under a microscope following various treatments (control, mono- and combination treatment) for a duration of five days. As depicted in Fig. 6d, the architecture of organoids exhibited a relaxed and even disintegrated state following treatment with TMZ and ABX combination. Moreover, the diameter of organoids was significantly reduced in the TMZ plus ABX group compared to other groups. The GBM PDOs from each group were collected and fixed for immunofluorescence staining of Ki-67 and γ-H2AX, respectively. Compared to the control and mono-drug treatment groups, the combination of TMZ and ABX significantly down-regulated Ki-67 expression and up-regulated γ-H2AX expression in organoids. This suggests that combination treatment can effectively inhibit organoid growth while inducing sustained DNA damage (Fig. 6e).

Finally, drug treatment sensitivity tests were performed on a cohort of GBM PDOs comprising 16 patients. The findings indicate that a combination treatment regimen comprising ABX and TMZ leads to an improved inhibition rate in over 80% of GBM cases (*n* = 13), with approximately 37.5% of GBM cases (*n* = 6) exhibiting a fold change in inhibition rate improvement exceeding 2 (Fig. 6f). The combination treatment group exhibited a significantly higher mean inhibition rate compared to the TMZ mono-treatment group (Fig. 6g). We also conducted an analysis on the correlation between genetic background and response rate in the PDOs cohort. Our findings indicate that GBM patients with IDH1/2 or ATRX mutation exhibit a nearly 100% response to combination treatment of ABX and TMZ (Fig. 6h), suggesting that this particular genetic profile may benefit from such a treatment regimen.

## Discussion

TMZ has been a standard chemotherapy drug for the treatment of glioblastoma for over 15 years. However, resistance to TMZ remains the primary cause of treatment failure in glioblastoma patients. Therefore, enhancing the sensitivity of glioma cells to TMZ has become a key focus of clinical researches. Currently, there is a lack of efficient chemo-sensitizers available in clinical practice. In this study, we demonstrate a synergistic effect of ABX and TMZ therapy in multiple experimental models of GBM, without any observed toxic side effects. In terms of molecular mechanisms, our findings indicate that ABX enhances the sensitivity of GBM to TMZ treatment by inducing sustained DNA damage and promoting ferroptosis. From a therapeutic perspective, combination therapy with TMZ and ABX demonstrates stronger synergistic anti-tumor effects compared to mono-therapy with TMZ alone in a prospective GBM PDOs cohort. The combination therapy of ABX and TMZ shows promise in treating GBM with reduced sensitivity to chemotherapy.

Paclitaxel (PTX), a microtubule-stabilizing agent, was initially identified as a potent therapeutic against glioblastoma multiforme (GBM) in preclinical models. Despite its high cytotoxicity against gliomas, the efficacy of PTX has not been fully exploited due to the protective blood–brain barrier (BBB). Studies have shown that PTX concentration can be detected in tumor tissue, but not in the surrounding brain parenchyma. This highlights the blood–brain barrier as a major obstacle to the effectiveness of PTX for infiltrative gliomas [20, 21]. Abraxane® is a novel albumin-bound formulation of paclitaxel that has recently emerged as an alternative to Taxol®. ABX is water-soluble and free of CrEL as a carrier. Daniel Y. Zhang et al. have demonstrated that ABX represented the optimal formulation for glioblastoma treatment due to its superior brain penetration and tolerability in comparison with traditional paclitaxel [14].

Previous research has demonstrated that the DNA damage response pathway can rapidly repair TMZ-induced DNA lesions in glioblastoma cells, potentially contributing to chemotherapy resistance [22]. However, DNA repair proteins can directly respond to and repair DNA damage through nuclear transport, thereby mitigating tumor injury from chemotherapy. The activation of NER pathway is a common cause of chemoresistance [23]. ERCC1 plays a pivotal role in the NER pathway, which is triggered by chemotherapeutic agents that induce DNA damage in cancer cells to effectively eliminate gliomas [24]. Sandra G. Boccard et.al have reported that targeting DNA repair genes, such as ERCC1, could serve as an adjuvant chemo-sensitization treatment in preclinical studies [25]. These results are in accordance with those found in our study. We observed that the addition of ABX based on TMZ significantly reduces ERCC1 expression and impedes the nuclear transport of ERCC1 protein. Additionally, an unexpected discovery was made regarding the reduction of XPC nuclear import, which is another crucial priming repair protein in the NER pathway. Steven Bergink et.al have reported that the XPC protein forms a supramolecular complex which recognizes and binds to sites of damaged DNA, thereby initiating the NER pathway [26]. Afterwards, our team has confirmed that the XPC protein can be transported into the nucleus by the cytoskeleton-related protein DHC2 as cargo, ultimately leading to TMZ-resistance in GBM cells [6]. In this study, we have also observed that ABX has the potential to resensitize GBM cells to TMZ chemotherapy by disrupting nuclear transport of XPC protein. The underlying mechanism may be attributed to ABX-induced cytoskeletal disruption and its impact on the molecular function of DHC2. Furthermore, it is noteworthy that the methylation status of cellular MGMT did not impact the efficacy of combination therapy of TMZ and ABX. We hypothesized that ABX, a novel microtubule-targeting agent, may disrupt the nuclear translocation process of MGMT protein in GBM cells with an unmethylated MGMT promoter. However, this needs to be further elucidated in future research.

In recent years, ferroptosis has garnered significant attention from researchers due to its potential implications in drug resistance mechanisms observed across various tumors [27–29]. As is widely recognized, the Xc-GSH-GPX4 system represents one of the most crucial cellular antioxidant defense mechanisms against ferroptosis [30]. Since GPX4 relies on GSH, hindering cystine uptake could potentially result in decreased intracellular GSH levels, reduced GPX4 activity and ultimately lead to an increase in ferroptosis [31]. RenXin Yi et.al discovered that dihydroartemisinin triggers ferroptosis in GBM by inhibiting GPX4, indicating the crucial role of GPX4 as a therapeutic target for GBM [32]. Furthermore, Meng-Yun Zhao and colleagues have reported that the combination of propofol and paclitaxel exhibits synergistic anti-cancer effects on cervical cancer cells [33]. On one hand, propofol and paclitaxel can activate the apoptosis pathway in cervical cancer cells; on the other hand, their combination can also induce ferroptosis by regulating the SLC7A11/GPX4 pathway [33]. In our study, we have observed that the combination therapy of ABX and TMZ can induce ferroptosis in GBM cells by regulating the GSH/GPX4 axis and HO-1 expression. Furthermore, ferrostain-1, a commonly used inhibitor of ferroptosis, was found to partially rescue ferroptosis in GBM cells. This suggests that other molecular mechanisms, such as impaired DNA damage repair, may also be involved. However, the molecular mechanisms underlying this crosstalk need to be further investigated in the future.

Tumor organoids, three-dimensional cell cultures derived from patient-derived tumor cells, hold great potential in advancing drug screening and predicting drug sensitivity for cancer treatment. Traditionally, the discovery of cancer drugs has heavily relied on two-dimensional cell line cultures and animal models. However, these models often fail to accurately reflect the heterogeneous genetic landscape and microenvironment of human tumors. Tumor organoids can serve as a valuable tool for predicting drug sensitivity, enabling the identification of patients who are most likely to respond positively to specific drugs. The researchers' establishment of a biobank containing 50 living colorectal cancer liver metastasis (CRLM) organoids has yielded promising results in predicting chemotherapy response [34]. Our research team has successfully established 16 cases of GBM patient-derived organoids (PDOs) and conducted sensitivity tests for drug combination therapy. The results indicate a significant improvement in the sensitivity of GBM patient-derived organoids to combination therapy with ABX and TMZ, which is also influenced by the molecular pathology of GBM. Notably, there exists a correlation between the molecular statuses of IDH and ATRX and the responsiveness of GBM PDOs to the combined drug treatment. The drug combination utilized in the study demonstrated efficacy in treating all GBM PDOs harboring concurrent IDH and ATRX mutations. Our findings suggest that the combination therapy of ABX and TMZ may enhance the sensitivity of TMZ chemotherapy, particularly in GBM subgroups with concurrent IDH and ATRX mutations.

This study demonstrates a synergistic effect of the drug combination ABX and TMZ in multiple GBM models, including the PDOs model. The corresponding mechanisms were also elucidated. It should be noted that this study is preliminary and certain aspects require further investigation. These limitations include the relatively modest sample size of tumor organoids employed in this study and the need to identify which molecular subtypes of tumors are most amenable to treatment with the ABX and TMZ drug combination. While no significant adverse events were observed, further validation in other animal models, including mammals and primates, as well as in human subjects is warranted.

## Conclusion

In summary, our findings suggest that ABX has the potential to enhance TMZ treatment sensitivity in GBM. The combined treatment of ABX and TMZ can induce sustained DNA damage by disrupting XPC and ERCC1 expression and nuclear localization. Additionally, the combination treatment can enhance ferroptosis through regulating HOXM1 and GPX4 expression. Our research provides a promising therapeutic strategy for GBM patients.

### Supplementary Information

**Additional file 1:** **Table S1.** Clinical and genetic characteristics of patients enrolled for the establishment of primary glioblastoma cells.

**Additional file 2:** **Figure S1.** The IC50 concentration value of TMZ (a) and ABX (b) in four GBM cell lines. **Figure S2.** TMZ and ABX combination index in four GBM cell lines was calculated by using the Chou–Talalay Index. **Figure S3.** GBM cell morphology changes were induced by prestimulation or combination of low dose ABX compared with TMZ alone. **Figure S4.** Cell viability of GBM cells with ABX pre-treatment for 24 h and followed with TMZ treatment at different concentrations. **Figure S5.** Evaluation of toxic side effects of drug combinations in vivo. **Figure S6.** Western Blot analysis showed the effect of ABX pre-stimulation on DNA damage in GBM cells. **Figure S7.** Top, change of γ-H2AX foci in GBM cells treated with TMZ (400 µM for U87-MG and G353; 800 µM for LN229 and G393) with or without low-dose ABX (12nM) for 48 h. Bottom, statistics on the number of γ-H2AX foci at various time points following drug elution. Scale bar, 10µm. **Figure S8.** Typical images of γ-H2AX (a), HO-1 (b) and GPX4 (c) expression immunohistochemical analysis in tumor tissue slices from GBM-bearing mice. Scale bar in (a), 50µm; scale bar in (b) and (c), 20µm. **Figure S9.** (a) Morphological changes of U87-MG cells treated with TMZ (400 µM) with or without low-dose ABX (12nM) for 72 h. (b) The expression of γ-H2AX between TMZ and TMZ+ABX groups by Western blotting. **Figure S10.** DNA damage response is not involved in ferroptosis-mediated efficacy of drug combination. **Figure S11.** Identification of PDOs related indicators.

**Additional file 3.** Supplementary Methods.

## Data Availability

The mass spectrometry proteomics data have been deposited to the ProteomeXchange Consortium ((http://proteomecentral.proteomexchange.org) via the iProX partner repository [35] with the dataset identifier PXD041176.
