# Correction: albumin-bound paclitaxel augment temozolomide treatment sensitivity of glioblastoma cells by disrupting DNA damage repair and promoting ferroptosis

**DOI:** 10.1186/s13046-023-02905-9

**Published:** 2023-11-23

**Authors:** Shanqiang Qu, Songtao Qi, Huayang Zhang, Zhiyong Li, Kaicheng Wang, Taichen Zhu, Rongxu Ye, Wanghao Zhang, Guanglong Huang, Guo-zhong Yi

**Affiliations:** 1 Department of Neurosurgery, Nanfang Hospital, Southern Medical University, Guangzhou, Guangdong People’s Republic of China; 2 The Laboratory for Precision Neurosurgery, Nanfang Hospital, Southern Medical University, Guangzhou, Guangdong People’s Republic of China; 3 Nanfang Glioma Center, Guangzhou, Guangdong People’s Republic of China; 4 Institute of Brain Disease, Nanfang Hospital, Southern Medical University, Guangzhou, Guangdong People’s Republic of China; 5 The First Clinical Medical College, Southern Medical University, Guangzhou, Guangdong People’s Republic of China

**Correction**: ***J Exp Clin Cancer Res*42, 285 (2023)**

10.1186/s13046-023-02843-6

Following publication of the original article [1], errors were identified by author in the Supplementary Figures, specifically:

- Supplementary Figure S8a - should be updated due to mislabeling issue. Author inadvertently used incorrect figures to depict the γ-H2AX expression of the G393 group Figure S8a.
- The figure legend of Figure S11 in Additional file 2: should be changed from ‘Figure 11’ to ‘Figure S11’

The corrections do not affect the overall result and conclusion of the article. The original article has been corrected. All the authors apologize for the mistakes in this manuscript.

### Electronic supplementary material

Below is the link to the electronic supplementary material.

Supplementary Material 1
