# Supplementary material for: Albumin-bound paclitaxel augment temozolomide treatment sensitivity of glioblastoma cells by disrupting DNA damage repair and promoting ferroptosis
Source: J Exp Clin Cancer Res. 2023 Oct 28;42:285. doi: 10.1186/s13046-023-02843-6 (PMC10612313; doi:10.1186/s13046-023-02843-6)
Supplement: Supplementary file 1 — Additional file 1: Table S1. Clinical and genetic characteristics of patients enrolled for the establishment of primary glioblastoma cells. [file 13046_2023_2843_MOESM1_ESM.docx]

**Table S1. Clinical and genetic characteristics of patients enrolled for the establishment of primary glioblastoma cells.**

| **Patient ID** | **Gender** | **Age** | **Pathologic diagnosis** | **Mutant genes** | **Wildtype genes** | **MGMT Promoter methylation** |
| --- | --- | --- | --- | --- | --- | --- |
| P16356 | Male | 32 | Primary GBM | TERT(c.1-124C>T) EGFR (p.G719S) | IDHI, IDH2, TP53, H3F3A HISTIH3B, BRAF, PIK3CA, | Unmethylated |
| P16393 | Male | 45 | Recurrent GBM | FANCD2(c.3963+2T>A) FGFR4 (c.1209 1212delCGCC p.A404Lfs* 46) IRF2(c.742-2A>T) PTEN(c385A>Gp.S129G) SOX17(c.972 977delGCACCA p. Q324_H325del) TBX3(c.1523C>T p.A508V) U2AF1(c.648+653delCGGTGG p.G217 G218del) | IDHI, IDH2, TP53, H3F3A HISTIH3B, BRAF, PIK3CA, | Unmethylated |
