# Supplementary material for: Albumin-bound paclitaxel augment temozolomide treatment sensitivity of glioblastoma cells by disrupting DNA damage repair and promoting ferroptosis
Source: J Exp Clin Cancer Res. 2023 Oct 28;42:285. doi: 10.1186/s13046-023-02843-6 (PMC10612313; doi:10.1186/s13046-023-02843-6)
Supplement: Supplementary file 2 — Additional file 2: Figure S1. The IC50 concentration value of TMZ (a) and ABX (b) in four GBM cell lines. Figure S2. TMZ and ABX combination index in four GBM cell lines was calculated by using the Chou–Talalay Index. Figure S3. GBM cell morphology changes were induced by prestimulation or combination of low dose ABX compared with TMZ alone. Figure S4. Cell viability of GBM cells with ABX pre-treatment for 24 h and followed with TMZ treatment at different concentrations. Figure S5. Evaluation of toxic side effects of drug combinations in vivo. Figure S6. Western Blot analysis showed the effect of ABX pre-stimulation on DNA damage in GBM cells. Figure S7. Top, change of γ-H2AX foci in GBM cells treated with TMZ (400 µM for U87-MG and G353; 800 µM for LN229 and G393) with or without low-dose ABX (12nM) for 48 h. Bottom, statistics on the number of γ-H2AX foci at various time points following drug elution. Scale bar, 10µm. Figure S8. Typical images of γ-H2AX (a), HO-1 (b) and GPX4 (c) expression immunohistochemical analysis in tumor tissue slices from GBM-bearing mice. Scale bar in (a), 50µm; scale bar in (b) and (c), 20µm. Figure S9. (a) Morphological changes of U87-MG cells treated with TMZ (400 µM) with or without low-dose ABX (12nM) for 72 h. (b) The expression of γ-H2AX between TMZ and TMZ+ABX groups by Western blotting. Figure S10. DNA damage response is not involved in ferroptosis-mediated efficacy of drug combination. Figure S11. Identification of PDOs related indicators. [file 13046_2023_2843_MOESM2_ESM.docx]

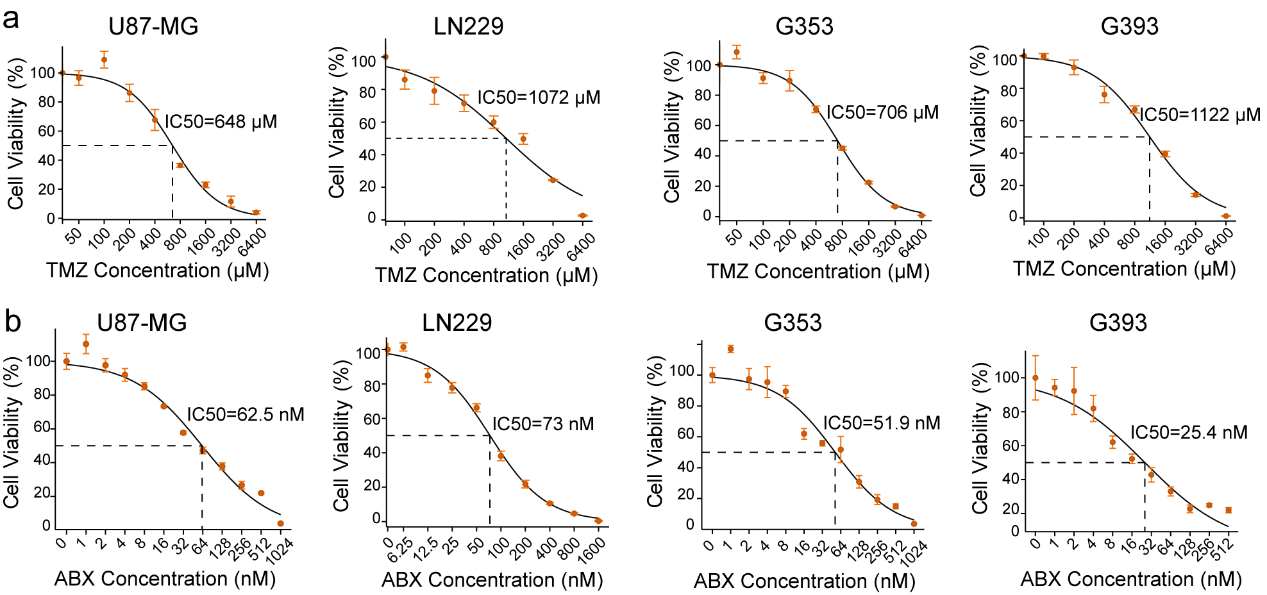


**Figure S1**

Figure S1. The IC_50_ concentration value of TMZ (a) and ABX (b) in four GBM cell lines.

**Figure S2**


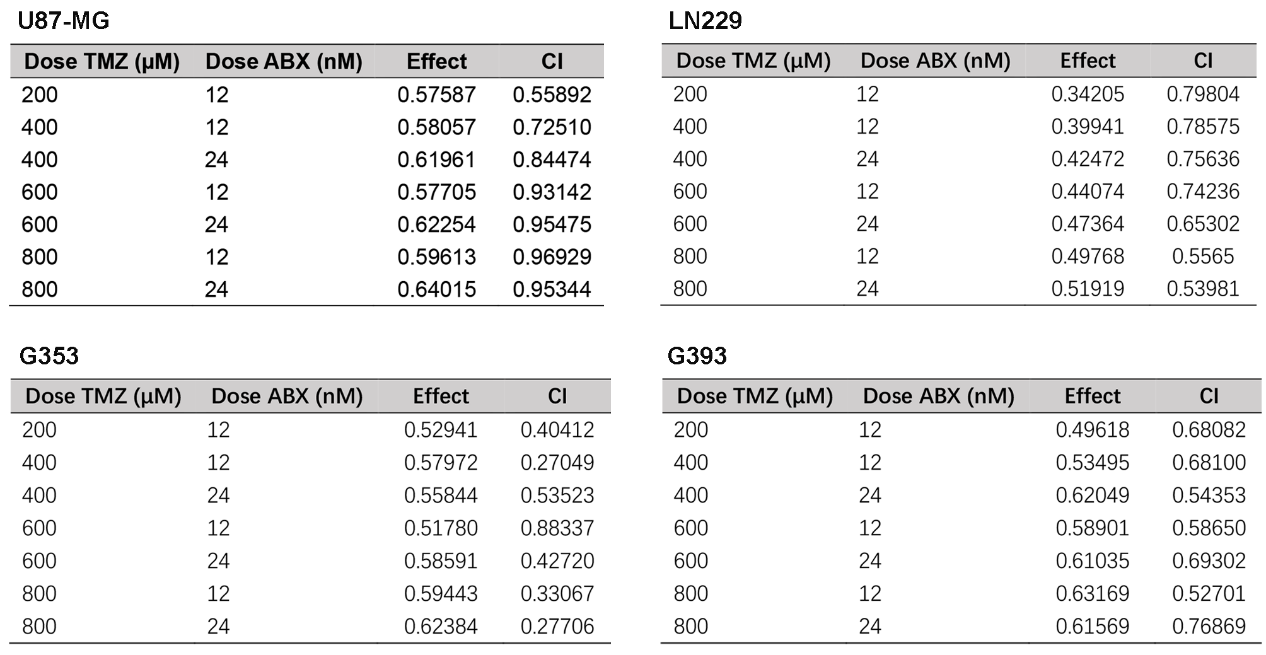


Figure S2. TMZ and ABX combination index in four GBM cell lines was calculated by using the Chou–Talalay Index.


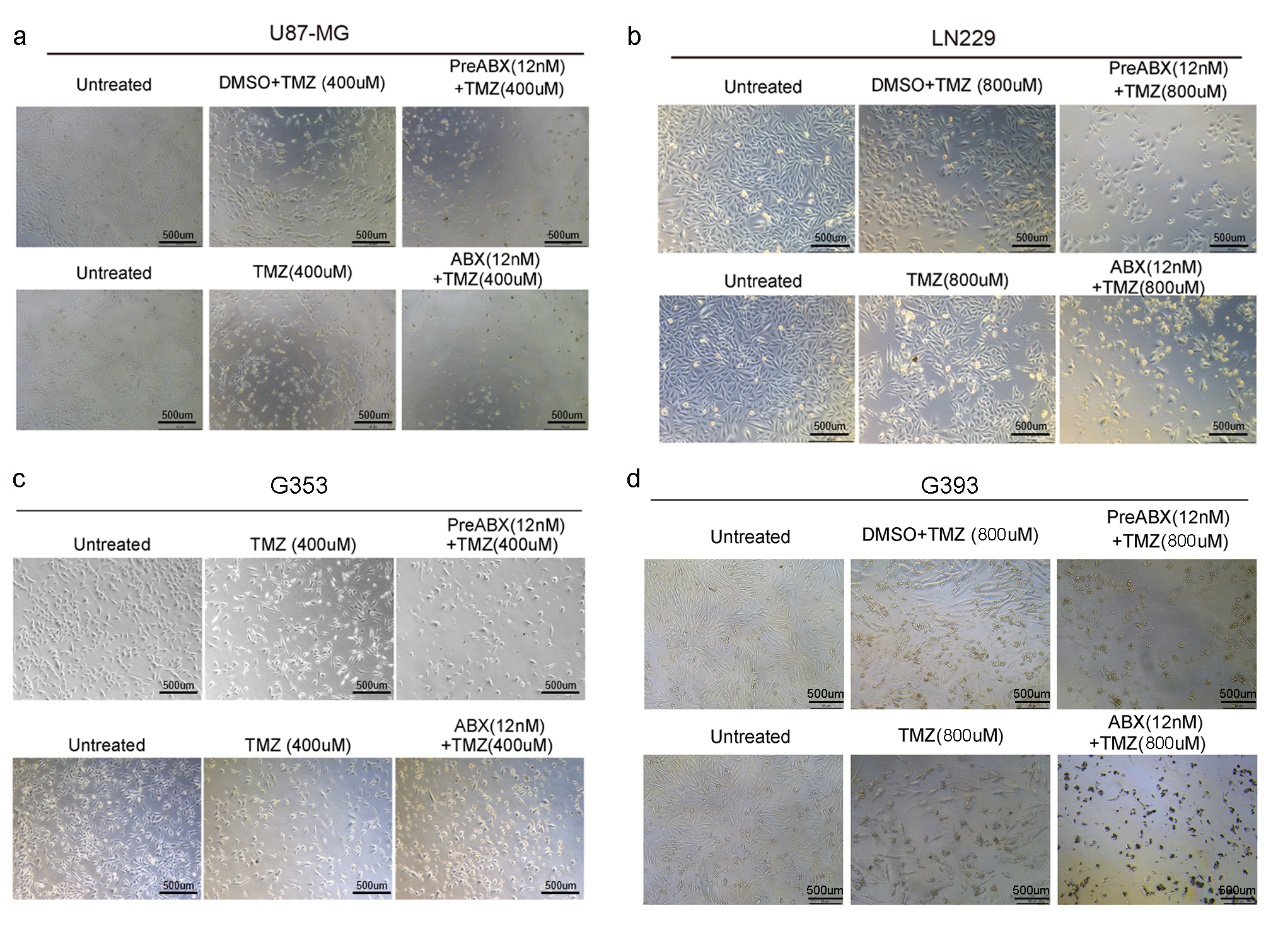


**Figure S3**

Figure S3. GBM cell morphology changes were induced by prestimulation or combination of low dose ABX compared with TMZ alone.

**Figure S4**


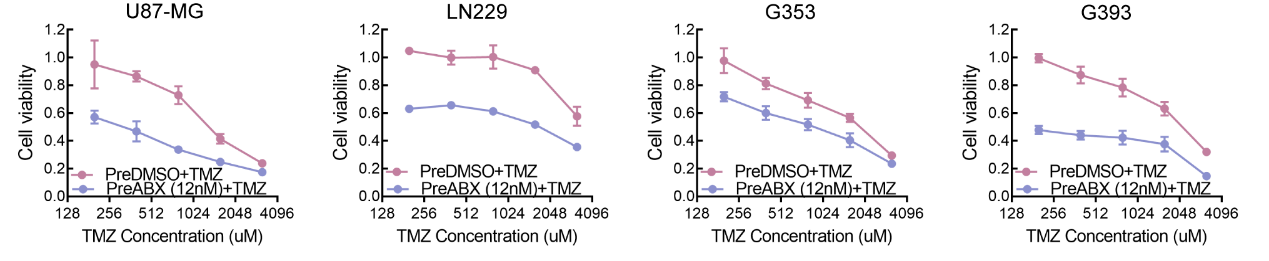


Figure S4. Cell viability of GBM cells with ABX pre-treatment for 24 h and followed with TMZ treatment at different concentrations.


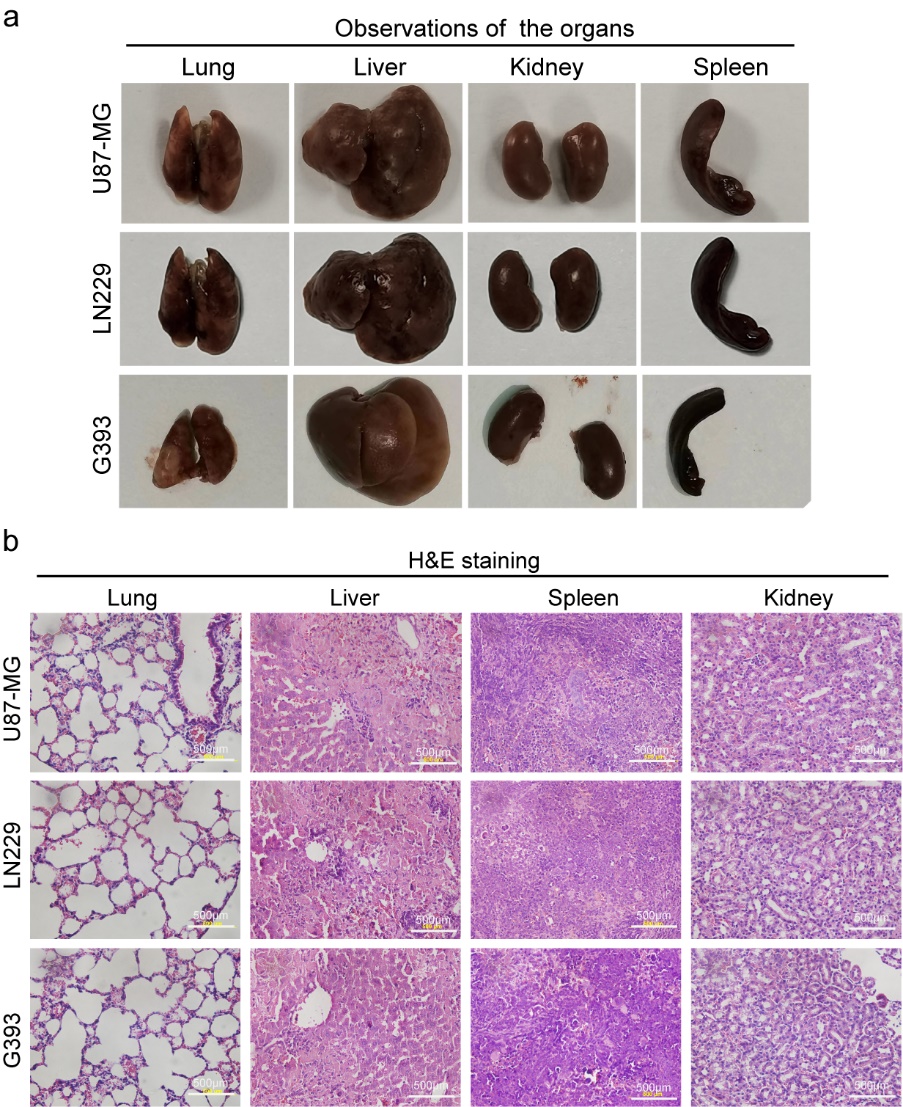


**Figure S5**

Figure S5. Evaluation of toxic side effects of drug combinations *in vivo*.

(a) The organs (e.g., lung, liver, kidney and spleen) that were harvested from the nude mice were photographed and are presented here. (b) H&E staining of different organs in nude mice. Scale bar, 400µm.

**Figure S6**


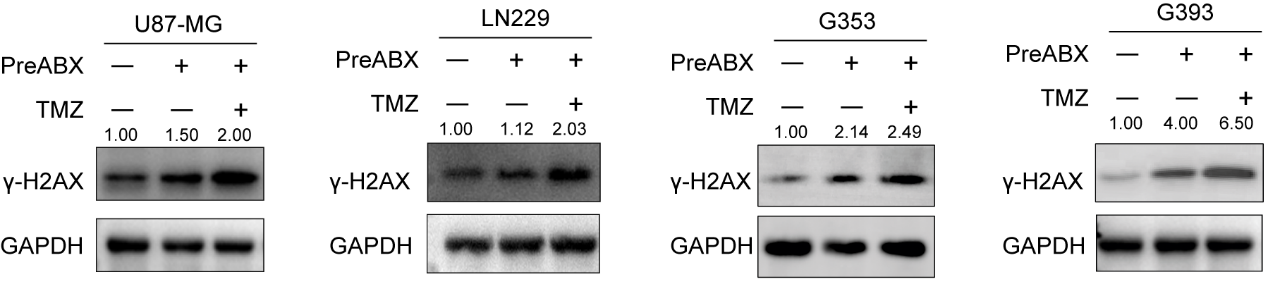


Figure S6. Western Blot analysis showed the effect of ABX pre-stimulation on DNA damage in GBM cells.


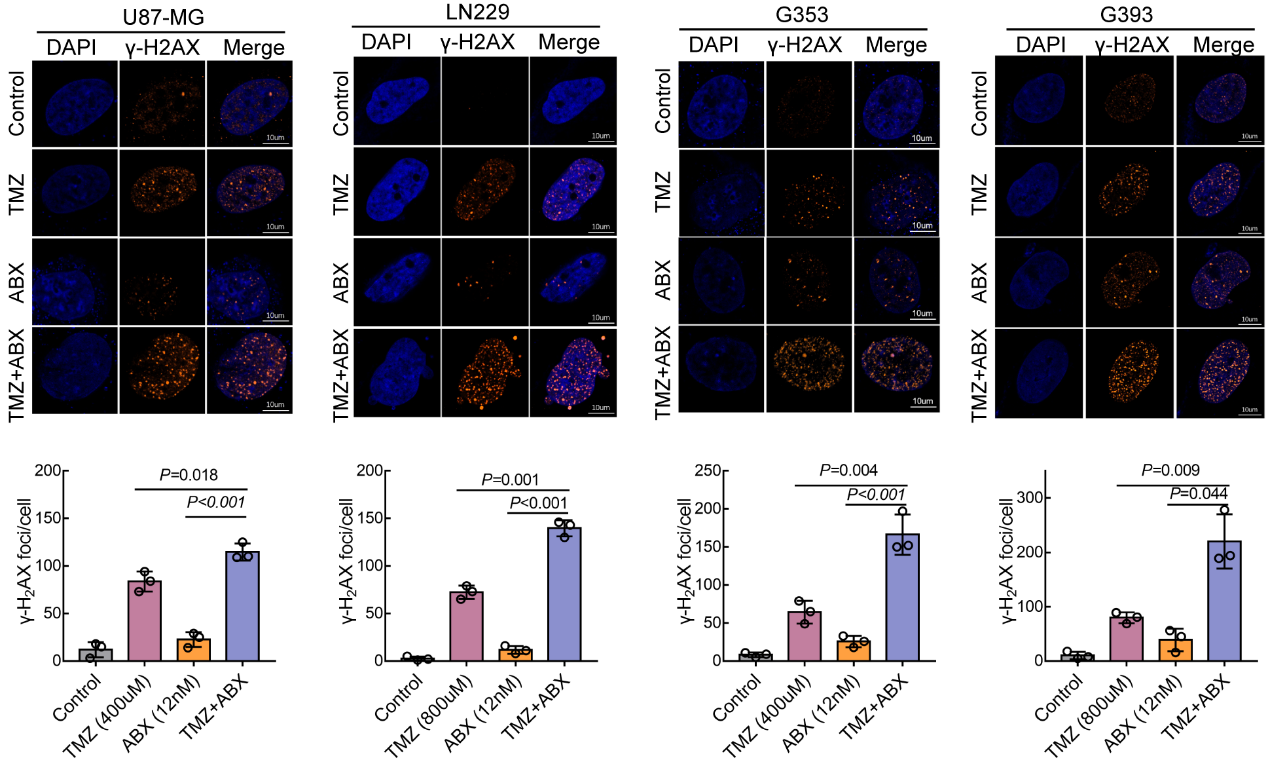


**Figure S7**

Figure S7. Top, change of γ-H2AX foci in GBM cells treated with TMZ (400 µM for U87-MG and G353; 800 µM for LN229 and G393) with or without low-dose ABX (12nM) for 48 h. Bottom, statistics on the number of γ-H2AX foci at various time points following drug elution. Scale bar, 10µm.

**Figure S8**


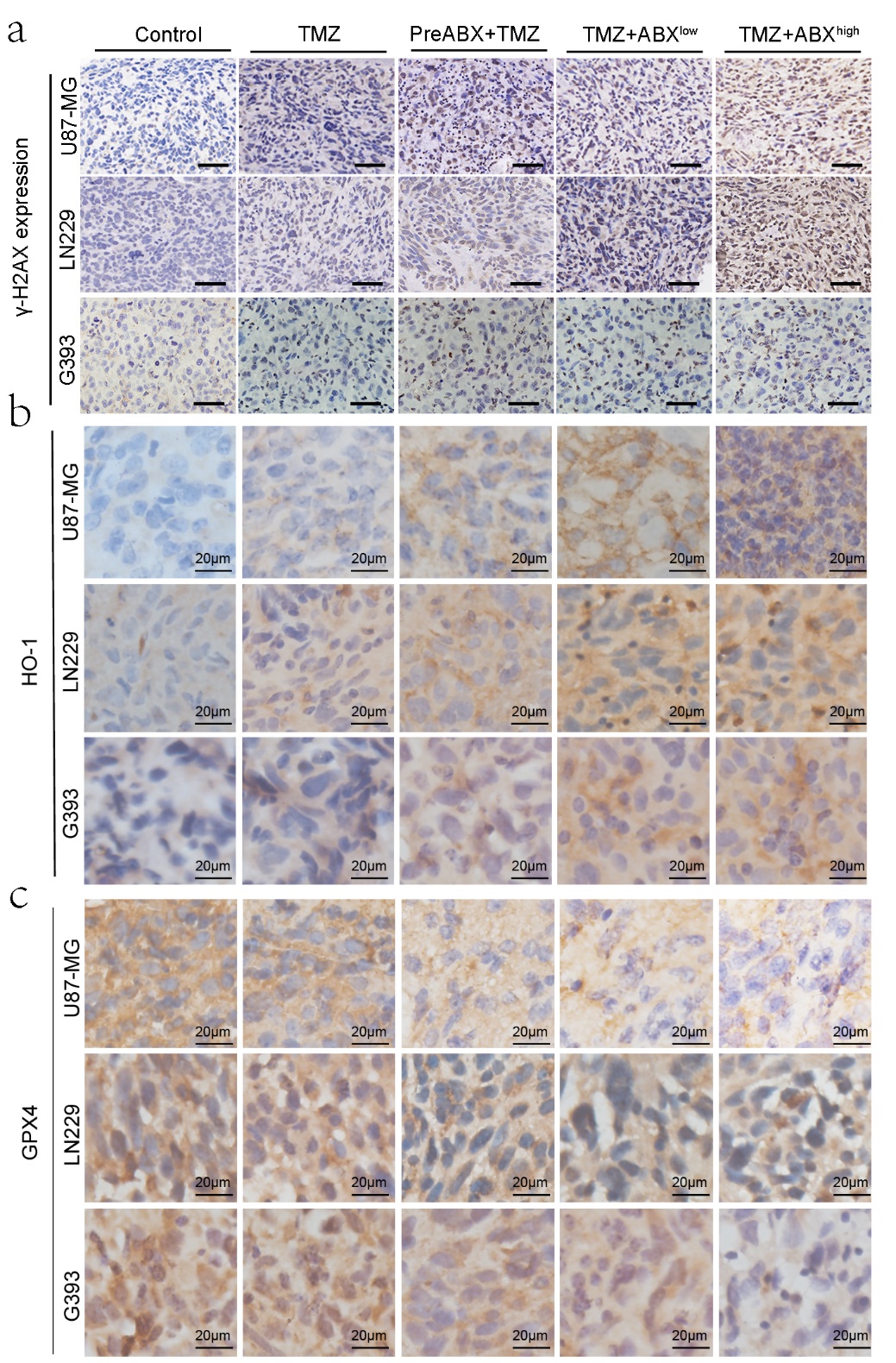


Figure S8. Typical images of γ-H2AX (a), HO-1 (b) and GPX4 (c) expression immunohistochemical analysis in tumor tissue slices from GBM-bearing mice. Scale bar in (a), 50µm; scale bar in (b) and (c), 20µm.

**Figure S9**


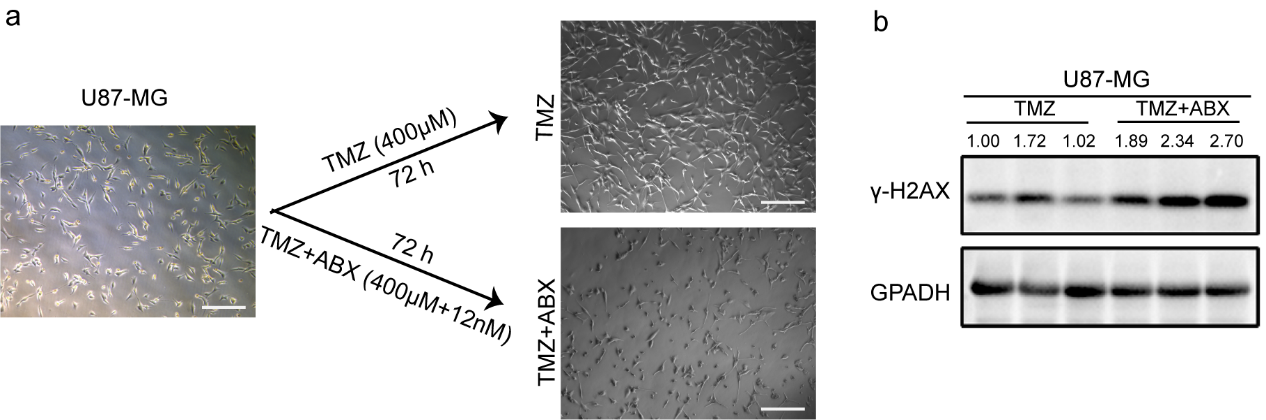


Figure S9. (a) Morphological changes of U87-MG cells treated with TMZ (400 µM) with or without low-dose ABX (12nM) for 72 h. (b) The expression of γ-H2AX between TMZ and TMZ+ABX groups by Western blotting.

**Figure S10**


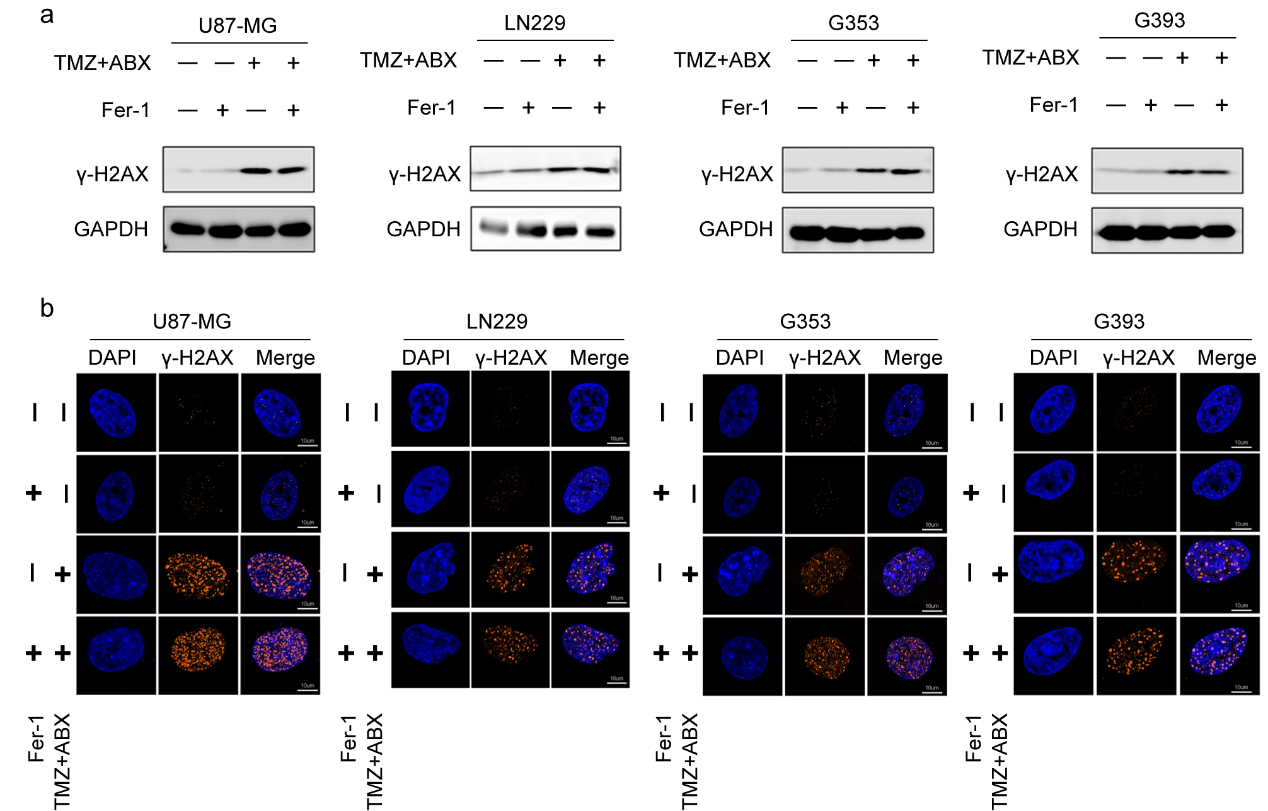


Figure S10. DNA damage response is not involved in ferroptosis-mediated efficacy of drug combination. (a) Protein levels of γ-H2AX were analyzed by western blotting in different groups with Fer-1 (2µM) or TMZ combination with ABX for 48 h. (b) Representative immunofluorescence images of γ-H2AX foci in GBM cell lines treated with Fer-1 (2µM) or TMZ combination with ABX for 48 h.

**Figure S11**


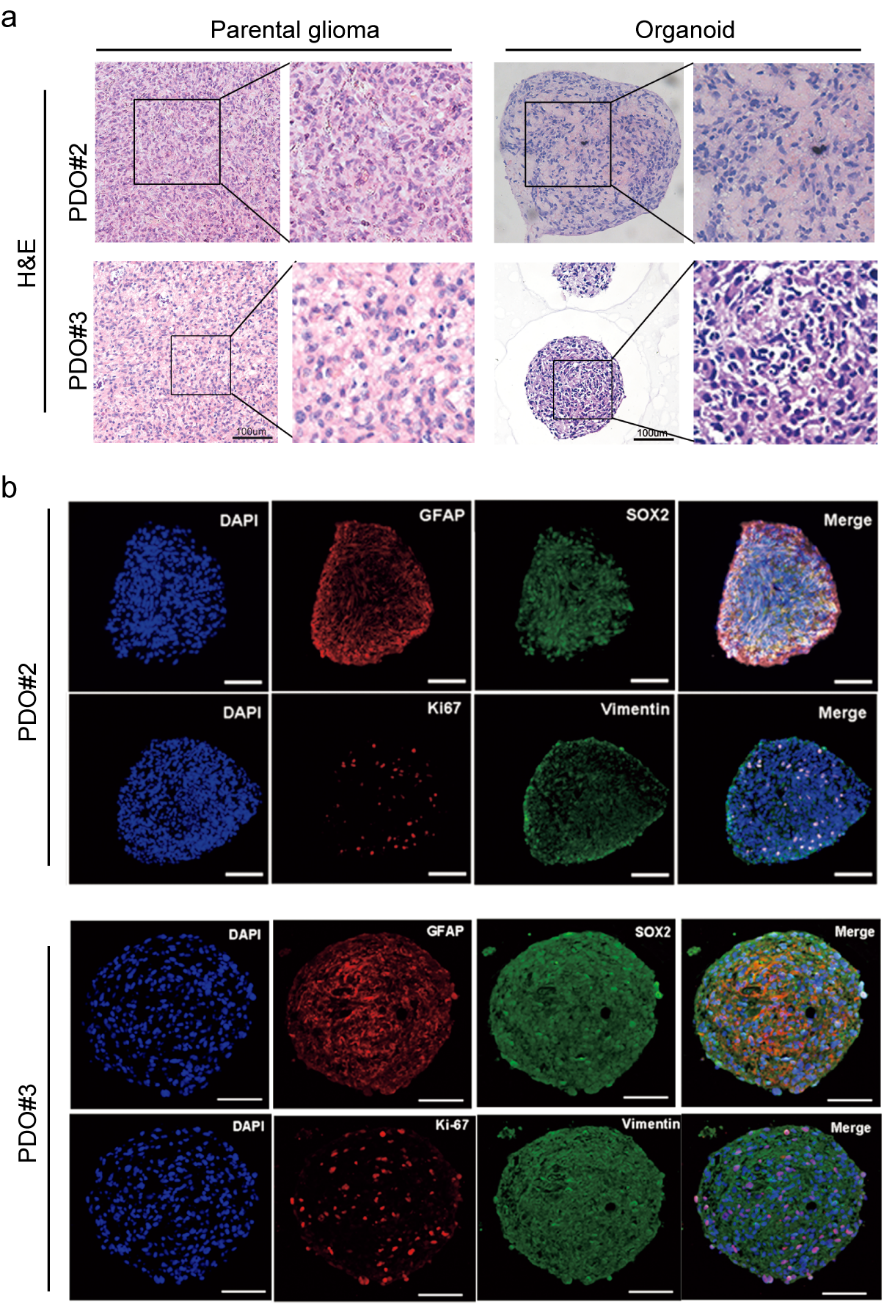


Figure S11. Identification of PDOs related indicators.

(a) Organoid sections and parental tumor sections were analyzed by H&E staining. (b) The GFAP, SOX2, Ki-67 and Vimentin markers were detected in PDO#2 and PDO#3 tissue slices by immunofluorescence staining.
