# Supplementary material for: Albumin-bound paclitaxel augment temozolomide treatment sensitivity of glioblastoma cells by disrupting DNA damage repair and promoting ferroptosis
Source: J Exp Clin Cancer Res. 2023 Oct 28;42:285. doi: 10.1186/s13046-023-02843-6 (PMC10612313; doi:10.1186/s13046-023-02843-6)
Supplement: Supplementary file 3 — Additional file 3. Supplementary Methods. [file 13046_2023_2843_MOESM3_ESM.docx]

**Supplementary Methods**

**Cell line and GBM samples**

The human U87-MG and LN229 cell lines used in this study were purchased from the American Type Culture Collection (ATCC). G353 and G393 are primary GBM cells isolated from fresh GBM tissues. These GBM samples were obtained from the Nanfang Glioma Center of Nanfang Hospital. All GBM cell lines were cultured regularly and underwent mycoplasma contamination tests ^6^. Briefly, cells were cultured in Dulbecco's Modified Eagle Medium (Cat.no. C3113-0500; Vivacell) supplemented with 10% fetal bovine serum (Cat.no. 04-001-1A; BI Biotech) and maintained at 37℃ in a 5% CO_2_ incubator. This study received approval from the ethics committee of Nanfang Hospital, Southern Medical University.

**Cell viability assay**

Cell viability evaluation was executed by the Cell Counting Kit-8 (CCK-8) assay, which was performed using CCK-8 kit (Cat.no. C6005; New Cell & Molecular Biotech). Briefly, we seeded around 3,000 cells into each well of 96-well plates (SORFA Life Science Research, Co., Ltd., Huzhou, China) (5 replicate wells per group) and incubated them at 37℃ for 24 h. Afterward, we switched the medium with fresh media supplemented with drugs and incubated for 48-72 h. Then, we replaced the medium in each well with 100 µL DMEM containing 10% of CCK-8 and allowed it to incubate for 2 h. Finally, we determined the absorbance at 450 nm using a microplate reader (Thermo Fisher, MA, USA).

**Colony formation assay**

For colony formation assay, 500-800 cells per well were seeded into 6-well plates (SORFA Life Science Research, Co., Ltd., Huzhou, China) and were incubated for 48 h to ensure adherence. Then, cells were treated with fresh medium containing the respective drug concentrations for around three days. Later, the medium was replaced with fresh growth medium and the incubation was continued for around three weeks. After three weeks, we washed the colonies with PBS thrice after discarding the medium. The colonies were fixed with 4% paraformaldehyde and stained with 0.4% crystal violet. After washing with PBS again, the colonies were then oven-dried and photographed under the camera.

***In vivo* imaging**

*In vivo* imaging was performed in intracranial glioma-bearing mice. Live *in vivo* imaging was performed at the Small Animal Imaging Facility (model no. Ami HT/ Ami HTX) at Nanfang hospital. For bioluminescent imaging, animals were anesthetized with isoflurane, injected intraperitoneally with 3 mg of D-Luciferin (Perkin Elmer) and imaged using IVIS Spectrum Imaging System. The Living Image software was used for analysis of the images post-acquisition.

**Immunohistochemistry (IHC)**

IHC assays were carried out on GBM samples or nude mouse tumor tissues to detect γ-H2AX and Ki-67 expression. Briefly, paraffin-embedded blocks were cut into 4-μm sections and deparaffinized and rehydrated. Antigen retrieval was performed by pressure cooking for 5 min in citrate buffer (pH=6.0), followed by blocking of endogenous peroxidase in 0.3% H_2_O_2_. After blocking with 5% bovine serum albumin (BSA) for 1 h, sections were incubated sequentially with primary antibodies and horseradish peroxidase-linked secondary antibody (Cat.no. SAP-9100; Zhongshan Golden Bridge Biotechnology Co., Ltd). The primary antibodies were anti-Ki-67 (Cat.no. ZM-0166; Zhongshan Golden Bridge Biotechnology Co., Ltd) and anti-γ-H2AX (Cat.no. ab11174; abcam). Sections were covered with diaminobenzidine for visualizing the staining and then counterstained with haematoxylin before being examined by microscope.

**Immunofluorescence**

For immunofluorescence, 5x10^3^ cells were grown on 20 mm confocal petri dishes. After 24h, cells were treated with fresh medium containing the respective drug concentrations and were further incubated for the indicated time at 37℃. Then, the culture medium was discarded and cells were washed three times with PBS. Cells were fixed for 10 min in 4% paraformaldehyde, permeabilized with 0.5% Triton™ X for 15 min and blocked with 5% BSA for 1 h. After removal of BSA, cells were incubated with the indicated primary antibody overnight. The primary antibody was removed the next day and washed with PBS. After washing with PBS, cells were incubated with fluorophore-conjugated secondary antibodies (Alexa Fluor® 488 or 555) in the dark for 45 min. after incubation, the second antibody solution was discarded and the cells were washed three time with PBS. After several washes, nuclei were counterstained with DAPI (Cat.no. ab228549; abcam). Images were captured on a Carl-Zeiss confocal microscope. Immunofluorescence staining of tissue sections can be performed according to the previously published protocol ^6^. The primary antibodies were anti-XPC (Cat.no. 14768S; CST), anti-γ-H2AX (Cat.no. ab11174; abcam), anti-Ki-67 (Cat.no. 9449S; CST), anti-Vimentin (Cat.no. BS-0756R; Bioss), anti-GFAP (Cat.no.3670S; CST), anti-SOX2 (Cat.no.3579S; CST), and anti-β-Tubulin (Cat.no. 86298S; CST).

**Comet assay**

The comet assay was performed with minor modifications as described ^15^. The steps were performed according to kit instructions (Cat.no. WLA123; WanleiBio, Shenyang, China). Briefly, cells were digested and proceed cell counting. Cells were then mixed with 0.5% low-melting-point agarose, plated on microscopical slides. Afterwards, cells were lysed for 2 h in lysis buffer (2.5 M NaCl, 100 mM EDTA, 10 mM Tris, 1% sodium lauryl sarcosinate, pH 7.5). The slides were transferred from solution to a horizontal electrophoresis apparatus at 20 V for 20 min. The samples were stained and placed under the fluorescence microscope for observation. Data was analyses using the Comet Assay Software Project (CASP software).

**LC-MS/MS analysis**

SDT(4%SDS，100mM Tris-HCl，1mM DTT，pH7.6) buffer was used for sample lysis and protein extraction. The amount of protein was quantified with the BCA Protein Assay Kit (Bio-Rad, USA). Protein digestion by trypsin was performed according to filter-aided sample preparation (FASP) procedure described by Matthias Mann. LC-MS/MS analysis was performed on a Q Exactive mass spectrometer (Thermo Scientific) that was coupled to Easy nLC (Proxeon Biosystems, now Thermo Fisher Scientific) for 60/120/240 min. The MS raw data for each sample were combined and searched using the MaxQuant 1.5.3.17 software for identification and quantitation analysis.

**qRT-PCR**

qRT–PCR experiments were performed as described previously ^16^. Briefly, the experiments were performed in a Lightcycler 96 (Roche) using the FastStart Essential DNA Green Master dye and polymerase (Roche). The final volume for each reaction was 10 μl, consisting of 5 μl of dye and polymerase (master mix), 2 μl of cDNA sample (diluted to an estimated 1-10 ng/μl equivalent of RNA) and 3 μl of the specific primer pairs. The primers used for the amplification of the indicated genes are listed as follows: GAPDH, forward 5’-CTCTTCCTTCCTTCCTTCCTTCCT-3’, reverse 5’-AGCACTGTGTTGGCGTACAG-3’ and PTGS2, forward 5’- CGGTGAAACTCTGGCTAGACAG -3’, reverse 5’- GCAAACCGTAGATGCTCAGGGA -3’

**Construction of PDOs**

Tumor tissues were obtained from patients undergoing surgical resection for GBM. GBM organoids were generated as previously described ^17^. Briefly, tumor tissues were washed well in sterile 0.9% NaCl solution supplemented with antibiotics and minced into small pieces. Afterwards, tumor fragments were digested with TrypLE (Fisher Scientific) for 45 min at 37℃. Digestion was terminated by dilutions with HBSS containing calcium and magnesium. The digest was filtered through a 100-μm filter. The cell clumps were washed two times with HBSS and resuspended in 5 ml of RBC lysis solution (MiltenyiBiotec). Cell clumps were washed twice with HBSS. After HBSS discard, the cell clumps were resuspended with Glioma Organoid Culture Medium (Accuroid, China, Cat. No. M112) and then were plated on six-well, low adhesion plated (Corning; Cat. No. 3471) on a rotary shaker at 37℃ in a humidified incubator with a 5% CO_2_ environment.
